# Physical Health Teaching for Inpatient Staff Quality Improvement Project

**DOI:** 10.1192/bjo.2026.11421

**Published:** 2026-06-30

**Authors:** George Lin, Aditya Raheja, Daim Meghwar, Nicola Ng, Alan Smith

**Affiliations:** Central and North West London NHS Trust, London, United Kingdom

## Abstract

**Aims:**

Cardiac arrest on ward–post incident review and reflections identified recognition and escalation of the physically deteriorating patient as a key improvement target to improve staff morale and confidence moving forward in context of incident.

SMART goals:

Specific – Improve nursing staff and allied health professionals’ ability in recognising, managing, and escalating physically unwell patients.

Measurable – with results measured via standardized questionnaire scores.

Achievable – through a specialized teaching programme developed by ward doctors.

Relevant – in context of recent significant incident on the ward.

Time specific – in a 6-month timeframe.

**Methods:**

Teaching sessions targeting initial assessment and escalation of the physically deteriorating patient were delivered on a two-weekly basis over a time of 4 months, each session was 30 minutes in duration. Subdivision of broad topics was introduced to address nuanced scenarios. Domains were chosen based on common clinical queries and situations that were identified on the ward from a survey of both doctors, nursing staff and allied health care professionals. Session content was pitched at a role-specific level. Session topics included–Airways, NEWS2, SBAR, hypoglycaemia, fluid monitoring, pain, seizures, stroke, UTI.

Data was collected via pre-/post-course questionnaires. The questionnaire consisted of 9 questions in SBA format, following a short clinical scenario stem – 1 question for each clinical domain. Confidence scores (Likert scale) were assessed for each of the 9 domains.

**Results:**

Confidence improved across all wards following the teaching sessions. Small but notable decline in clinical performance post-training in some areas. Persistent performance gap between RMNs and HCAs.

Teaching sessions were delivered in modality of one live session per topic – making it difficult to ensure that all surveyed staff had attended all sessions. There were limited opportunities for staff to review the teaching material after a session, or especially if a session was missed due to rota or clinical commitments.

Therefore an opportunity was identified to develop e-learning modules focusing on weakest domains (Airways, NEWS, SBAR, UTI, hypoglycaemia).

**Conclusion:**

Mode of delivery would be changed to e-learning modules on a digital learningplatform. This allows for easy access for staff in their various schedules, the ability to revisit the material, as well as increased ease in collecting data, adjusting the teaching materials and maintaining consistency in quality of teaching across time.

